# Rationale and Methodology of The PopulatION HEalth and Eye Disease PRofile in Elderly Singaporeans Study [PIONEER]

**DOI:** 10.14336/AD.2020.0206

**Published:** 2020-12-01

**Authors:** Preeti Gupta, Ryan Eyn Kidd Man, Eva K Fenwick, Amudha Aravindhan, Alfred TL Gan, Sahil Thakur, Bao Lin Pauline Soh, Joanne M Wood, Alex A Black, Angelique Chan, David Ng, Teoh Khim Hean, Edwin Goh, Chong Foong-Fong Mary, Jenny Loo, Ciaran Gerard Forde, Charumathi Sabanayagam, Ching-Yu Cheng, Tien Yin Wong, Ecosse L Lamoureux

**Affiliations:** ^1^Singapore Eye Research Institute and Singapore National Eye Centre, Singapore.; ^2^Duke-NUS Medical School, Singapore.; ^3^Singapore Institute of Technology, Health and Social Sciences, Singapore.; ^4^Queensland University of Technology, Brisbane, Australia.; ^5^National Dental Centre, Singapore.; ^6^Saw Swee Hock School of Public Health, National University of Singapore, Singapore.; ^7^National University Hospital, Singapore.; ^8^Singapore Institute for Clinical Sciences, National University of Singapore, Singapore.; ^9^Department of Ophthalmology, National University of Singapore, Singapore

**Keywords:** epidemiology, aging, eye disease, visual impairment, visual function system, population-based study

## Abstract

To describe the rationale, design and methodology of a geographically-representative and population-based study investigating the epidemiology, impact, personal and economic burden of age-related eye diseases, declining visual and other sensory systems in Asians aged >60 years in Singapore.PIONEER (The PopulatION HEalth and Eye Disease PRofilE in Elderly Singaporeans Study) is currently a cross-sectional study targeting 3152 Chinese, Malay and Indian adults who are Singapore citizens or permanent residents aged 60 years and older living across Singapore. The study is intended to be longitudinal, with several waves of data planned to be collected in the future. The sampling frame consisted of 7000 names derived from age, gender and ethnicity-stratified random sampling of individuals >60 years. Selected individuals were invited via letters, home visits, and telephone calls for a clinical assessment at the Singapore Eye Research Institute. Individuals with limited mobility were examined in a custom-designed mobile eye clinic. Questionnaires were subsequently administered at participants’ homes by trained interviewers in their preferred language. A total of 3,299 participants (from East, West, North and South Singapore) were approached from December 2017 to November 2019. Of these, 953 (28.5%) were deemed ineligible. Out of 2,346 eligible participants, 904 (38.5%) refused, and 1,442 (61.5%) attended our clinical testing protocol, giving an initial response rate of 61.5%. Of these, 1,170 (81%) were cognitively able to complete the questionnaire assessment. The mean age±SD of our participants was 73.8±8.6 years; n=798 (55.3%) were female; and 828 (57.4%) were of Chinese ethnicity. The findings from this study will allow a deeper understanding of the risk factors and impact of aging in Asian populations, particularly in relation to the visual function and other functional system.

Population ageing is a global phenomenon, with 1.5 billion people estimated to be aged ≥60 years worldwide by 2030 [1]. However, 60% of these people will be living in Asia [2]. Globally, Singapore has the world’s highest average life expectancy at 85 years, and 1 in 4 people will be aged over 65 years within the next two decades [3-5]. This ageing phenomenon is expected to lead to a corresponding increase in the number of older people in Singapore living with age-related eye disorders, as the prevalence of these diseases increases dramatically after 70 years of age [6].

The exact impact of aging on the prevalence of eye diseases in Asian societies is unclear. Although several Asian population-based studies [7-21] have provided information on the epidemiology of eye diseases, most have recruited “middle-aged (40-50 years)” and “young-old (55-65 years)” participants, with a lack of information in those aged >75 years. For example, our Singapore Epidemiology of Eye Diseases (SEED) Study, had <10% of its participants aged >75 years [22, 23]. Furthermore, previous studies have primarily focussed on measuring visual acuity (VA), with little information on other components of the visual function system such as contrast sensitivity [CS], stereo-acuity [SA], colour vision [CV], and field of view, which are strongly age-related and play critical roles in independence, navigation in the community, activities of daily living and quality of life (QoL) [24-26]. Moreover, while previous epidemiological studies have been ‘eye-centric’, the ageing of the eye does not happen in isolation. For instance, age-related eye diseases occur concurrently with other comorbidities and declines in other functional systems [27]. Therefore, a more holistic understanding of the deterioration in the components of the ageing visual system, in conjunction with age-related changes in other functional systems such as body composition, sensory, dental, and bone health, in elderly Singaporeans is imperative to inform public health policy and resource allocation planning. Unfortunately, such data are currently unavailable in Asian populations.

To bridge this knowledge gap, we implemented a nationally representative and population-based study of Chinese, Malays, and Indians aged ≥60 years, entitled “The PopulatION HEalth and Eye Disease PRofilE in Elderly Singaporeans (PIONEER).” PIONEER aims to: (a) determine the epidemiology of age-related ocular pathologies, vision impairment (VI), and associated changes in various components of the ageing visual function system (primary outcome); (b) evaluate the cross-sectional and longitudinal relationships between age-related eye diseases, VI, the ageing visual function system and nutrition, other sensory losses (hearing and smell), oral health, cognition, objective body composition indices and multimorbidity; and (c) elicit the comprehensive economic, clinical and patient-centred impact of age-related eye diseases, VI and the ageing visual function system on the individual and caregiver.

In this paper, we provide a detailed description of the methodology of the PIONEER study.

## MATERIALS AND METHODS

### Study Design and population

PIONEER is currently a cross-sectional, population-based and epidemiological study of Chinese, Malay and Indian adults who are Singapore citizens or permanent residents aged 60 years and older living across Singapore. The study is intended to be longitudinal, with several waves of data planned to be collected in the future. The study commenced in December 2017 and follows the principles of the Declaration of Helsinki, with ethics approval obtained from the SingHealth Centralized Institutional Review Board (CIRB, Reference #2016/3089). All participants are given a choice to provide their written, informed consent in either Chinese, Malay, Tamil or English. The study protocol and rationale are explained to participants in their preferred language including, common Singaporean dialects such as Hokkien, Cantonese, Teochew and Hakka, as well as common Indian languages, including Hindi, Punjabi etc. by trained study coordinators proficient in these languages/dialects.

PIONEER assessment comprises two sessions including (1) a comprehensive clinical examination conducted at Singapore Eye Research Institute (SERI), or on a mobile eye clinic, a custom built bus outfitted with standardized clinic equipment; and (2) an extensive questionnaire protocol subsequently administered at the participant’s home in his/her preferred language, by trained interviewers. The cognitive status of the participants is assessed using the 6 item Cognitive Impairment Test (6-CIT) [28] before proceeding with the study procedures. For those who score ≥ 8 in 6-CIT (indicating mild cognitive impairment or worse) during the recruitment visit, the study details are explained to, and verbal consent obtained from, the primary caregiver. Furthermore, on the day of the clinical examination, research coordinators made sure to obtain written consent from the caregiver in the presence of an impartial witness, before commencing study related procedures. A copy of the signed consent form was given to the participant/caregiver per our study protocol. Those with 6-CIT scores <8 (no cognitive impairment) undergo the full (clinical and questionnaire assessment) protocol, whilst individuals with cognitive impairment undergo only the clinical assessment.

### Sampling Frame

Of the 5.8 million resident population in Singapore, ethnic Chinese (74.4%), Malays (13.4%) and Indians (9.0%) make up the majority of the population. Given that other ethnic groups (Eurasians, Caucasians, Japanese, Filipino and Vietnamese) contribute to only ~3.2% of its population, we chose to focus only the three main ethnic groups in PIONEER. We used criteria set by the Singapore census to define ‘Chinese’, ‘Malay’ and ‘Indian’ ethnicities. This definition includes all persons of Chinese, Malay or Indian origin, as indicated on the National Registration Identity Card, which is provided to all Singapore citizens and permanent residents. We have used this definition in our previous population-based studies in Singapore [29-31].

In 2017, a geographically representative sample of 7000 Singaporeans aged >60 years (stratified by age, gender, and ethnicity, and based on the estimated 2016 population distribution) residing across Singapore was obtained from a national database which have been used in previous population-based studies [32], to participate in the PIONEER Study. Individuals of Malay and Indian ethnicity, females, and older age groups were oversampled to ensure enough respondents for analyses in these groups.

**Figure 1.**
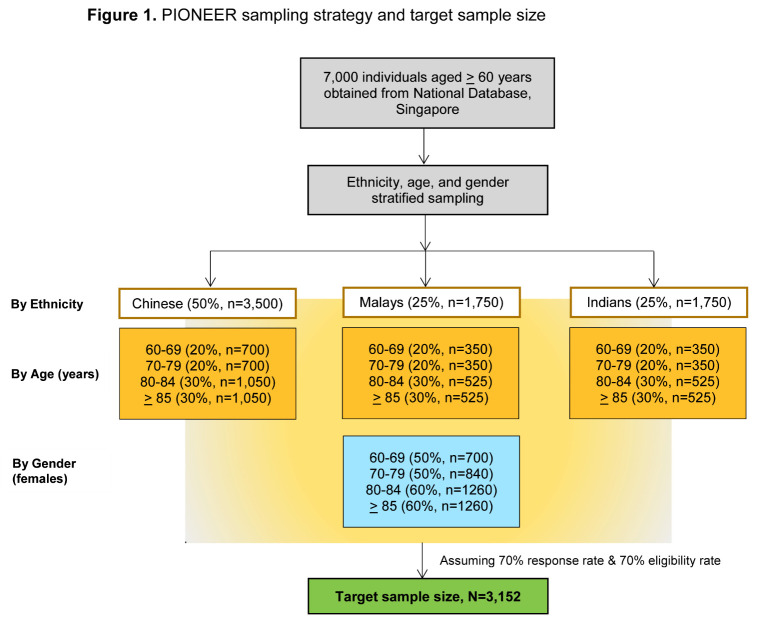
PIONEER sampling strategy and target sample size.

### Sample Size and Power

The sample size needed to estimate the prevalence of VI and major age-related eye diseases (cataract, refractive error, presbyopia, age related macular degeneration, glaucoma, diabetic retinopathy) - with 95% confidence intervals not wider than 4% ranged from N=707 for glaucoma to N=1936 for VI. Prevalence estimates were obtained from an analysis of participants aged above 70 years from a local population-based study, the Singapore Epidemiology of Eye Diseases study [33]. To have an adequately powered sample for subgroup analyses involving stratification by age, gender, ethnicity and visual function components (CS, SA, and CV), we increased the target sample size from 1936 to 3152 individuals. This sample size is adequate for estimating the prevalence of our secondary outcomes such as hearing loss, mobility, falls, cognition, sarcopenia, frailty, and independent living with the same level of precision as our primary outcomes. Assuming an eligibility rate of 70% and a response rate of 70%, we estimated that a sampling frame of 7000 names would be sufficient to achieve our target sample size.

**Figure 2.**
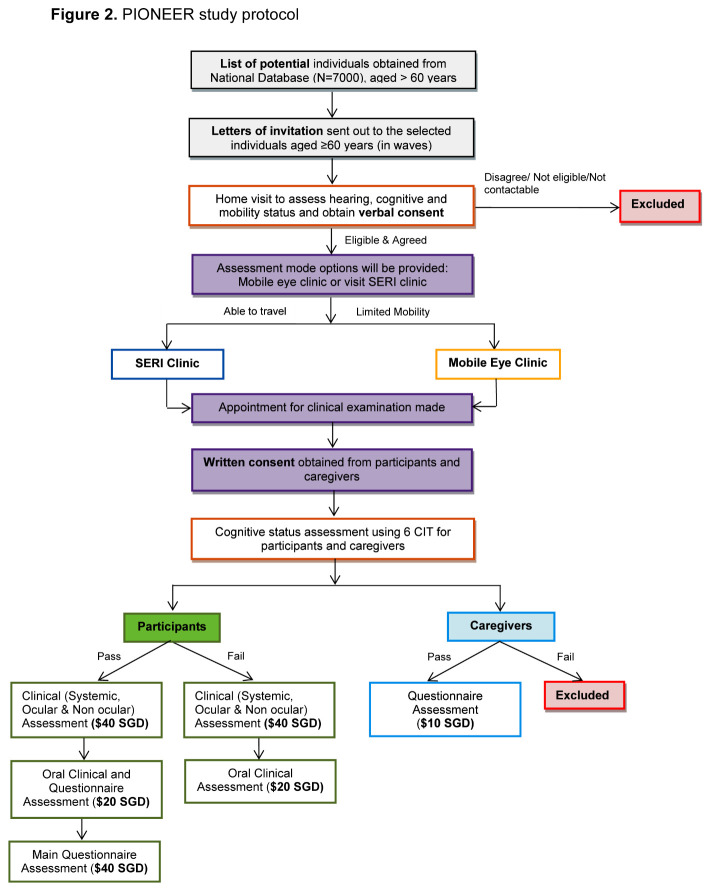
PIONEER study protocol.

We performed a single-stage stratified random sampling of individuals based on ethnicity sampling fractions of 50%, 25% and 25% for Chinese, Malays and Indians, respectively, i.e. 3500, 1750, 1750. Within each ethnic group, sampling fractions were 20%, 20%, 30% and 30% for the age groups 60-69y, 70-79y, 80-84y, and ≥85y, respectively (700, 700, 1050, 1050 for the four age groups in Chinese; 350, 350, 525, and 525 in Malays and Indians). Lastly, female sampling fractions were 50%, 50%, 60%, and 60% for the age groups 60-69y, 70-79y, 80-84y, and ≥85y, respectively. The corresponding estimates for the final sample size of 3152 stratified by ethnicity, gender and age are shown in Figure 1.

We over sampled minority ethnic groups (Malays and Indians), females, and older age groups, to increase the precision of estimates in these smaller subgroups. As such disproportionate sampling skews the distribution of the overall sample in terms of age, gender and ethnicity from the national distribution, individuals have to be weighted according to the resulting probabilities of being selected so that aggregated estimates are calibrated in line with the national distribution in terms of age, gender and ethnicity. For example, more weight will be given to males since their selection probabilities have been reduced relative to females, while less weight will be given to Malays and Indians as their selection probabilities have been inflated relative to Chinese when sampling from the national pool. We will define these sampling weights using the statistical method of post-stratification by aligning age, gender and ethnicity distribution to that of the national sampling frame. Similarly, not all sampled individuals will eventually participate, and participants may differ systematically from non-participants in terms of the distribution of age, gender and ethnicity (we will unfortunately not be able to collect other information on non-participants as we had to comply with Singapore’s Personal Data Protection (privacy) Act). Depending on how large these differences come to be, we will also consider applying weights for non-response following the same principles for calculating sampling weights as described above.

### Eligibility Criteria and Recruitment Strategies

All 7000 names are initially considered eligible to participate. Therefore, an invitation letter outlining the study details and inviting the addressee and caregiver to participate in the study is sent to all individuals on the list, in batches. This is followed by a visit to their home by the study recruitment officers to ascertain eligibility and agreement to participate. A potential participant is considered “ineligible” if he/she is uncontactable, in prison, residing in nursing home, deceased or terminally ill, bedridden or otherwise unable to give informed consent (e.g. due to dementia, severe hearing impairment, or muteness). Following an on-site eligibility assessment by the recruitment officer of a potential participant’s hearing (self-reported), cognitive (6-item Cognitive Impairment Test) [28], and mobility (self-reported) status, all eligible individuals are provided with printed and verbal information on the examination procedures and associated benefits and risks. Thereafter, if the individual is interested, verbal consent is obtained, and a clinic appointment is made. If the participant is absent during the home visit, a letter is left to encourage him/her to contact the study team. A potential participant is termed “not contactable” after six unsuccessful telephone calls and four home visits each conducted at different days (including weekdays and weekends) and times (morning, afternoon and evening). Individuals on the sampling list who declined the initial invitation are contacted again at a later time in a further attempt to recruit him/her, with at least one contact made every 3 months. The PIONEER recruitment strategies are shown in Figure 2.

For those who agree to participate, a reminder telephone call is made a day prior to the scheduled appointment to remind participants to attend. An individual who misses his/her appointment will have the appointment rescheduled. On the day of their appointment, study participants are requested to bring along their national identity card, and habitual distance and near spectacles. In addition, they are advised to refrain from wearing contact lenses, if worn, in order to avoid potential distortions and aberrations of the cornea arising from contact lens wear. As part of the informed consent process, our recruitment and study officers are trained to ensure all potential participants are aware that participation in the study is entirely voluntary and that they have the right to withdraw from the study or any specific clinical assessments at any time. Our clinical testing protocol takes on average ~3.5 hours. In cases where our elderly participants are unable to complete the tests during the same visit, another appointment is made within 1 month from the first visit with the participant’s consent, to complete all the remaining clinical assessments, along with the questionnaire administration. Furthermore, we offer free transportation and pick-up services to our subjects, and following the successful completion of the assessments, the participant is reimbursed for his/her time.

Regardless of the cognitive status of individuals who agree to participate, their primary caregiver is also contacted to ask for his/her willingness to be interviewed to assess the quality of life impact of caring for elderly relatives from the carer’s perspective. Caregivers also complete the 6-CIT to assess cognitive functioning before written informed consent is taken, with those who fail being excluded from this portion of the study.

**Figure 3.**
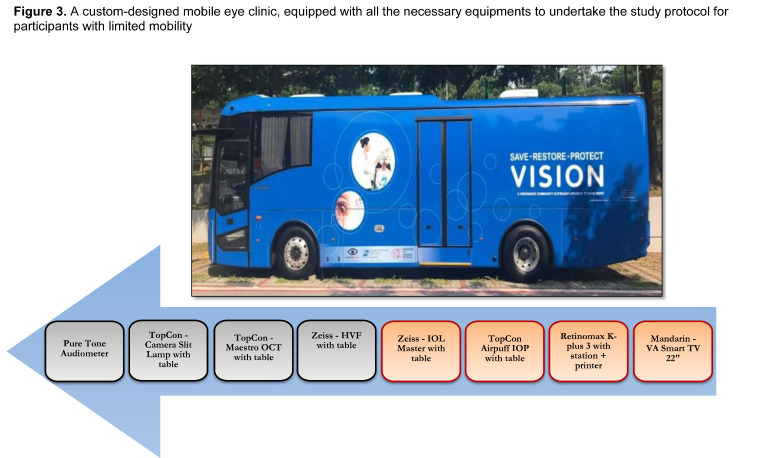
A custom-designed mobile eye clinic, equipped with all necessary equipments to undertake the study protocol for participants with limited mobility.

### Clinical Examination (systemic, ocular, and non-ocular)

**Figure 4.**
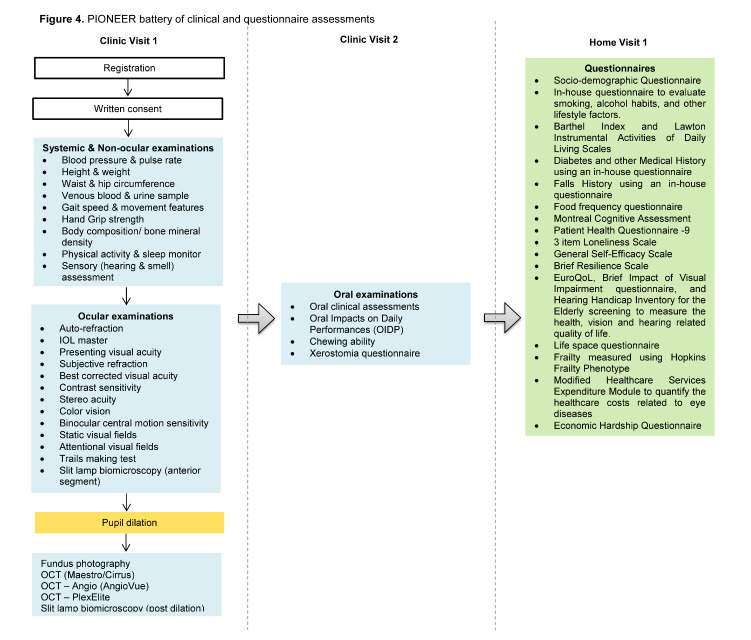
PIONEER battery of clinical and questionnaire assessments.

The clinical assessment component of PIONEER is conducted at SERI. For those participants with limited mobility (e.g. wheelchair bound), we offer the option to undergo study testing in a custom-designed mobile eye clinic, equipped with all the necessary equipments to undertake the study protocol (Fig. 3). At the study clinic, participants undergo a comprehensive battery of standardised examination procedures (Fig. 4), including:

′ Physical (height, weight, waist and hip circumference, body mass index-BMI, and blood pressure-BP)
′ Ocular (visual function assessment including VA, CS, SA, CV, standard visual fields and attentional field of view, slit lamp, gonioscopy, pupil dilation, fundus photography and optical coherence tomography imaging)
′ Non-ocular (gait speed, grip strength, body composition and bone health, physical activity, frailty, hearing, smell identification, and dental assessment)
′ Laboratory components (non-fasting venous blood and urine sample)

### Assessment of the Components of the Visual Function System

*Distance Visual Acuity (presenting & best corrected) and subjective refraction*: Monocular and binocular presenting distance visual acuities (PDVA) are assessed using a LogMAR (Log of Minimum Angle of Resolution) chart (Lighthouse International, Distance VA Number Chart, CAT No. C102) with the participant wearing his/her habitual prescription under photopic conditions (85 cd/m^2^) at 4m. If the participants are unable to read the largest line of letters on the VA chart, the chart is moved to 2m. However, if he/she is still unable to make out any lines at 2m, finger counting, hand movement and the ability of the eye to perceive light with a pen torch are assessed. Binocular measurements are made with both eyes viewing the target. Monocular and binocular best corrected distance VA (BCDVA) is then assessed using a trial frame and lenses under the same photopic conditions as that of PDVA measurements. Using the auto-refraction results as a starting point, the refinement of the sphere, cylinder and axis is performed until the BCDVA is obtained. Both PDVA and BCDVA are scored on a letter by letter basis with each letter worth 0.02log units.

*Near Visual Acuity:* Binocular presenting near VA (PNVA) is performed under photopic conditions at 40cm with the participant’s habitual correction. The same PDVA and BCDVA methodology is used to assess PNVA, namely the last line attempted where ≥ 3 mistakes are made, combined with the number of mistakes made on previous lines, are used to calculate a letter-by-letter logMAR PNVA score. Binocular best corrected near VA (BCNVA) measurement is conducted after BCDVA assessment, with participants’ best corrected prescription for the testing distance. Plus sphere trial lenses are utilized to obtain BCNVA measurements binocularly. The spherical dioptric correction is recorded along with the corresponding BCNVA.

*Contrast Sensitivity* (CS) (ability to recognise targets of different levels of contrast [faintness]) is measured using Pelli-Robson Contrast Sensitivity Chart as described elsewhere [34]. In brief, CS is measured monocularly and binocularly with the participant’s best distance correction, corrected for the testing distance of 1 m with a +0.75D working distance lens under photopic condition. Scores range from 0.00 to 2.25 logCS with higher values indicating better CS.

*Stereo-Acuity* (ability to see in 3-D) is assessed using the Frisby Stereo test [35]. Participants are presented with a stereo image on a sequence of three transparent plates, and are asked to identify the circle that has the depth cue in one of four squares for each of the three plates at a distance of 60 cm while wearing best corrected near correction. Scores range from 40-150 arc sec with lower values indicating better SA.

*Colour Vision* is measured using the Farnsworth D-15 test. Participants are asked to arrange 15 different colour discs in a sequential colour series, monocularly, while wearing best corrected near correction. Abnormal colour vision is defined as one or more major crossings, where the difference between two adjacent caps is more than three steps in the better-eye.

### Other Ocular Assessments

*Binocular Central Motion Sensitivity:* Motion sensitivity is assessed binocularly with habitual distance correction at a 3.2 m testing distance. Participants report the perceived direction of movement of a group of small targets generated on a computer screen (which move in one of four directions) and the minimum amount of stimulus movement that is correctly perceived is recorded as a displacement threshold, in logDegArc.

*Attentional Field of View (AFoV):* AFoV is a functional test of binocular visual processing speed for rapid detection and localization of central and peripheral targets under conditions of divided and selective visual attention (absence and presence of visual distractors, respectively). The test assesses higher order cognitive abilities, but performance also relies on visual sensory function since targets must be visible in order to be detected. The test has demonstrated high levels of reliability and validity [36, 37]. The test is conducted on an iPad, where participants identify a central task (direction of a large high-contrast tumbling E) and simultaneously identify the location of a peripheral target (a low-contrast Gabor patch). Processing speeds are calculated as the minimum presentation time (in ms) at which participants correctly identify both the central and peripheral targets. Faster speeds indicate better visual processing speeds.

*Neuro-psychological test of Visual Attention Trail Making Test (TMT):* Trail-making A and B tests are pen and paper tests which participants complete binocularly with their habitual near correction. In Part A, participants are asked to draw lines to connect in ascending order a series of randomly positioned circles containing numbers (1-2-325), as quickly as possible. In Part B, the circles include both numbers and letters, and participants are required to draw lines to connect the circles in an alternating ascending order between the numbers and letters (1-A-2-B-..13-L), which requires additional executive function processing during the visual search task. The time to complete both tests is recorded in seconds.

### Non-ocular Assessments

*Gait speed* is assessed with participants walking a distance of 4 m (15 feet) at their usual speed. Scoring of the gait speed test is in seconds. A score of less than 0.8 m/s is defined as slow gait speed [38].

*Gait features using ZurichMOVE Sensor:* The ZurichMOVE Sensor provides gait parameters during a typical walking session, including walking speed (m/sec), dual limb support time (sec; the duration of time when both feet are in contact with the ground during walking), stride time (sec; the duration between consecutive strides), stride length (m; the distance between consecutive strides), step time (sec), step length (m), and minimum toe clearance (m; the minimum distance with respect to the ground during the swing phase of gait) [39]. Participants are asked to walk for a minimum of 2.5 minutes to a maximum of 6 minutes. The variability of dual limb support time is linked to balance control, while variability in stride time provides rhythmicity. Similarly, variability of step length and time provide coordination and variability in minimum toe clearance is associated with obstacle avoidance [40-42]. The variability in these parameters over the entire period of walking will provide the gait features.

*Hand Grip Strength:* Participants’ grip strength (in kilograms) in the dominant hand is measured three times, with the participant seated and elbow flexed at 90 degrees, with a rest period of 30 seconds between each measurement. The average of the three readings will be utilised in analyses.

*Objective Physical Activity and Sleep Monitor:* Physical activity and sleep are monitored using an ActiGraph accelerometer (GT9X band). Participants are instructed to wear the watch on the non-dominant wrist over a period of 7 consecutive days (24 hours a day, except during water-based activities) and maintain a sleep log. The watch is collected by the study coordinator on the 8^th^ day. Raw acceleration data, number of steps taken, physical activity intensity (mild, moderate or vigorous), sleep latency, total sleep time and sleeping efficiency data are retrieved from the watch.

*Body Composition & Bone Mineral Density* measures are assessed using dual energy X-ray absorptiometry (DXA; Hologic Discovery-W; Hologic Inc, Bedford-MA), which is a non-invasive imaging modality utilizing very low dose X-rays (~0.005 to 0.01 millisievert). DXA quantifies participants’ body composition profiles, including fat and muscle mass, and bone mineral density (for entire single hip, and lumbar spine) measures. Standard patient preparation and procedural protocol are followed. The examinations are performed by an Allied Health Professions Council accredited radiographer to ensure accuracy in positioning and delineation of bone map and region of interest.

A detailed description of the clinical protocol, and definitions of ocular and systemic diseases are shown in Supplementary Table 1.

### Questionnaire Administration

During home interviews, all questionnaires are digitally administered using a tablet to participants who pass the 6-CIT in order to collect data on the following outcomes:

′ Demographic (age, gender, marital status)
′ Socio-economic (education, family income, housing, occupation)
′ Lifestyle (smoking, alcohol consumption, physical activity)
′ Self-reported history of systemic and eye diseases, medication use, and surgical history
′ Family history of systemic and eye diseases
′ History of falls and fractures
′ Cognitive function [43]
′ Depression [44]
′ Loneliness, social isolation [45], and behaviour (self-efficacy) [46], [47]
′ General health [48], vision [49], and hearing related QoL [50]
′ Independence (activities of daily living [ADL] [51], nstrumental activities of daily living [IADL]) [52]
′ Frailty [53]
′ Nutrition [54]
′ Economic impact [55] and household economic hardship [56]
′ Oral health [57]
′ Adult carer QoL [58]

A detailed description of the questionnaire protocol is provided in Supplementary Table 2. All questionnaires capturing patient reported outcomes, including, cognition [59], depression [60], functional status (ADL, IADL) [61, 62], social isolation [63], quality of life (general, vision and hearing related) [64-66], frailty [67] and oral health [57] have been previously validated in our local population studies.

### Quality Assurance and Control

All study officers including interviewers and recruitment officers are required to undertake Research Ethics and Compliance Training (an online program for research ethics and compliance education). They are trained by qualified personnel and are required to demonstrate competency in the relevant procedures and questionnaire administration before being certified to perform these procedures and conduct interviews on participants. Study informed consent and all questionnaires are translated into Mandarin, Malay and Tamil languages, and then back translated into English, by a certified translation company. Furthermore, the translated versions are proof-read to ensure the meaning is consistent with the original. A detailed checklist outlining the study inclusion/exclusion criteria is given to the recruitment officers to conduct proper eligibility testing. A pilot study (N=20; 5 of each language) was conducted prior to commencement of the main study to test out PIONEER recruitment strategies and workflow and ensure that all staff are familiar with the examination procedures. The quality of the data collected is checked periodically by the key investigators. Quality control test for BMD is performed everyday using Hologic Anthropomorphic Spine Phantom. Automatic Body Composition Calibration and radiographic uniformity test are done on a weekly basis. All study instruments are serviced annually (or as specified) to ensure accuracy in measurements.

### Data Storage

Data are collected in paper and digital formats via the research electronic data capture software (REDCap, Singapore). Clinical examination records, questionnaire responses, printouts (e.g., autorefraction, intra-ocular lens [IOL] master parameters), and biochemistry results are compiled into participant-specific case report forms that are labeled with the participant’s unique study number. Imaging data, including digital fundus, retinal imaging, and DXA images and analyses report are retrieved directly from the imaging equipment and stored in their respective computers, identifiable only by the study number, date created, file path, format, and size. The password protected database is sent to a dedicated data management team for implementation of data management procedures including quality checks and further data cleaning. Retinal photographs are stored in digital format for grading. Images are backed up on to the external drive and then securely transferred on to the SERI server. This storage method ensures there are two sets of copies in different formats. Finally, monthly meetings are conducted between data collection staff, the project manager (PG) and the principal investigator (EL) to discuss discrepancies identified during data collection.

### Statistical Analyses

Analyses will be performed using the commercially available STATA statistical software (Version 15, StataCorp, College Station, Texas). In future analyses, age- and gender-standardized prevalence rates will be calculated for major age-related eye diseases, VI, low vision, and presbyopia. Regression analyses under the generalized linear models’ framework will be used to:

1 Compare visual system components between those with and without specific eye diseases adjusted for traditional risk factors (i.e. hypertension, diabetes, cardiovascular disease).
2 Determine the associations between multimorbidity, multimorbidity-related factors (such as multiple treatment modalities, polypharmacy and the burden of disease management such as multiple daily drops for glaucoma and monthly injections for retinal diseases) with prevalent eye diseases and components of the ageing function system. A summary multimorbidity score will be created based on totaling the number of comorbid conditions and analyzed both as a continuous score and as a categorical variable (0-1, 2-4, ≥5).
3 Determine the associations between body composition measures (e.g. fat and muscle mass) with age-related eye diseases.
4 Determine the associations between major eye diseases and VI, and cognitive impairment, psychosocial wellbeing, and vision specific QoL data.

Where eye-specific analyses are concerned, the approach of generalized estimating equations will be used to estimate marginal associations that take into consideration the correlation between the two eyes of an individual. The economic impact of eye diseases, VI and the ageing visual function system, will be assessed by separately presenting estimates of direct and indirect costs by key stratification variables, including age, gender and ethnicity.

## RESULTS

A total of 3,299 participants (from East, West, North and South Singapore) were approached from December 2017 to November 2019. Of these, 953 (28.5%) were deemed ineligible. Out of 2,346 eligible participants, 904 (38.5%) refused, and 1,442 (61.5%) attended our clinical testing protocol, giving an initial response rate of 61.5%. Of these, 1,170 (81%) were cognitively able to complete the questionnaire assessment.

**Table 1:** Age, gender and ethnicity of participants (n=1442) vs. non-participants (n=904).

Mean ± SD or n (%)			
	Participants n = 1442	Non-participants n = 904	P
Age	73.8 ± 8.6	77.6 ± 8.7	<0.001
Gender			
Male	642 (44.5%)	357 (39.5%)	0.015
Female	798 (55.3%)	547 (60.5%)	
Ethnicity			
Chinese	828 (57.4%)	673 (74.5%)	<0.001
Indian	420 (29.1%)	165 (18.3%)	
Malay	160 (11.1%)	66 (7.3%)	

A substantial proportion of our sample was deemed ineligible to participate in the study. This was because our sampling frame only contained the individual’s name, age, gender, ethnicity, contact address and his/her regional residential zone. Participants’ cognitive, hearing and mobility status, and other health conditions (e.g., bedridden) were ascertained only at the home visit screening. Likewise, the status of participants residing in nursing home or prison was only discovered after the home visit screening.

Table 1 compares the age, gender and ethnicity data of non-participants (n=904) versus participants (n=1442). Compared to participants, non-participants were older, more likely to be female and Chinese. However, we could not compare other sociodemographic data of non-respondents with respondents in order to comply with Singapore’s Personal Data Protection (privacy) Act.

## DISCUSSION

PIONEER is an elderly- and systemic-centric, population-based study of Chinese, Indians and Malays. The study is ideally placed to investigate the clinical, biological, anthropometric, and psychosocial phenotypes of contemporary elderly Singaporeans living with vision loss and other sensory impairments to better understand the magnitude, consequences, burden, and complex mechanisms underlying ageing. Upon completion and with information from all ethnic groups, PIONEER will likely contribute to new and much needed data of the burden associated with age-related sensory decline in Singapore to inform public health initiatives; and assist policy and decision makers in planning health care availability, utilization, and resource allocation. Moreover, PIONEER will likely inform researchers and clinicians about the economic burden and QoL impact associated specifically with the ageing visual function system as well as multimorbidity more generally.

The main strengths of PIONEER include its extremely well-characterized participant sample, with high-quality ocular and systemic data, and a rich collection of bio-samples. The examinations in PIONEER are standardised and reproducible with a strong emphasis on quality control. Use of standardised protocols will enable direct comparison, validation, and pooling of analyses with other population-based studies. Furthermore, PIONEER focuses exclusively on older participants (≥ 60 years), a sector of our population that has not been well studied previously and will thus offer novel insights into the world of an elderly individual. The current study has some limitations. First, our detailed clinical testing protocol of ~3.5 hours resulted in fatigue in some of our elderly participants. However, it is unlikely to affect our data quality as we followed a randomized testing order and our participants were given appropriate breaks during the testing process. Moreover, we have split our clinical protocol into two visits for subjects who cannot manage the long testing protocol. Second, as the questionnaire protocol was only administered to the participants who passed the 6-CIT, we do not have patient-reported outcomes data on cognitively impaired individuals. Third, our initial recruitment rate is slightly low (~61.5%). This is likely because telephone numbers for our participants were not available (due to the Personal Data Protection Act regulation in Singapore), resulting in home-based (as opposed to telephone-based) recruitment, which is an extremely time consuming and inefficient recruitment method. However, we have adopted several measures to improve the participation rate of our study, such as increasing the number of recruitment officers, offering free transportation and pick-up services to our subjects, and providing printed information (such as brochures) on the study procedures and their associated benefits and risks at the point of recruitment. The current response rate is ~65%.

In summary, PIONEER employs a methodical elderly- and systemic-centric approach to provide novel insights into how the visual function system changes with age, its interaction with nutrition, sensory measures, oral, mental, and bone health, body composition and multimorbidity; as well as the consequences and burden of these multi-morbid visual changes. Furthermore, PIONEER comprehensively evaluates the impact of eye diseases, VI, and ageing visual function from a patient-centred perspective, supply information on the burden of eye disease and multimorbidity from the caregiver’s perspective and quantify the societal costs and personal economic hardships associated with VI. Such data will generate an evidence-based cost-effectiveness model needed to assess the economic benefits of interventions and treatment modalities in this segment of the Singapore population. Information from this study will be critical for the planning of public health strategies for screening, early diagnosis and intervention in various ocular and systemic diseases, translatable not only to Singaporeans but to other Asian populations outside Singapore.

## Supplementary Materials

The Supplemenantry data can be found online at: www.aginganddisease.org/EN/10.14336/AD.2020.0206.
