# Supplementary material for: Rationale and Methodology of The PopulatION HEalth and Eye Disease PRofile in Elderly Singaporeans Study [PIONEER]
Source: Aging Dis. 2020 Dec 1;11(6):1444–58. doi: 10.14336/AD.2020.0206 (PMC7673841; doi:10.14336/AD.2020.0206)
Supplement: Supplementary file 1 — The Supplemenantry data can be found online at: www.aginganddisease.org/EN/10.14336/AD.2020.0206. [file AD-11-6-1444-s.pdf]

SUPPLEMENTARY DATA

Rationale and Methodology of The PopulatION HEalth and Eye Disease PRofile in Elderly Singaporeans Study [PIONEER

Preeti Gupta[1], Ryan Eyn Kidd Man[1], Eva K. Fenwick[1,2], Amudha Aravindhan[1], Alfred TL Gan[1], Sahil Thakur[1], Bao Lin Pauline Soh[3], Joanne M Wood[4], Alex A Black[4], Angelique Chan[2], David Ng[2], Teoh Khim Hean[5], Edwin Goh[5], Chong Foong-Fong Mary[6], Jenny Loo[7], Ciaran Gerard Forde[8], Charumathi Sabanayagam[1,2], Ching-Yu Cheng[1,2,9], Tien Yin Wong[1,2,9], Ecosse L. Lamoureux[1,2,9*]

© 2020. Gupta P et al. Published online at http://www.aginganddisease.org/EN/10.14336/AD.2020.0206

SUPPLEMENTARY DATA

Supplementary Table 1. Clinical (anthropometry, biochemistry, ocular and non-ocular) assessment protocol and definitions of ocular and systemic diseases in PIONEER.

Clinical Outcomes	Equipment used	Assessment technique	Definition of Outcomes or Diseases
**Anthropometry**			
Height	Wall-mounted measuring tape	Height is measured in cm	Body mass index (BMI) is determined as (Weight[kg]/Height[m]^2).<br><br><u>Generalized obesity</u> is based on Asian-defined BMI cut-points [75], obese: BMI ≥30 kg/m^2
Weight	Calibrated digital scientific weight scale (SECA, model 782 2321009; Vogel & Halke, Germany)	Weight is measured in kg	
Waist and hip circumference	A non-stretchable medical tape	Waist circumference (WC) is taken at the smallest horizontal girth between the costal margins and the iliac crests at the end of tidal expiration, while hip measurement is made at the maximal protuberance of the buttocks. The waist-hip ratio (WHR) is calculated as waist divided by the hip circumference.	<u>Abdominal obesity</u> is defined based on Asian WC categories [76], central obesity in men: WC ≥ 90 cm; women: WC ≥ 80 cm
Blood pressure (BP; systolic & diastolic) and pulse rate	A digital automatic blood pressure monitor [77] (Dinamap model Pro Series DP110X-RW, 100V2; GE Medical Systems Information Technologies, Inc., USA)	BP is taken with the participant seated and after 5 minutes of rest. It is measured on two occasions, 5 minutes apart. If the readings differ by more than 10 mmHg (systolic) or 5 mmHg (diastolic), a third reading is taken. The BP of the individual is then taken as the mean between the two closest readings.	<u>Hypertension</u>: Systolic BP ≥140mmHg, diastolic BP ≥90mmHg, physician diagnosis [78], use of BP medication and/or self-report
**Biochemistry Tests**			
Venous blood sampling, DNA archival, and urine sampling	Blood and urine samples are processed and analysed at Quest Laboratories Pte Ltd (Singapore) for measurement on the same day. The lab is accredited by The College of American Pathologists' Laboratory Accreditation Program (AU-ID: 1377543).	Venepuncture is conducted by a trained phlebotomist and 27 ml of non-fasting venous blood is collected to determine non-fasting levels of serum lipids (total cholesterol, high density lipoprotein cholesterol (HDL) and low density lipoprotein cholesterol (LDL), and triglycerides), glycated Haemoglobin (HbA1C), creatinine, albumin, random glucose, C-reactive protein, calcium, vitamin D, full blood count, and blood typing.<br><br>Serum is also collected for DNA extraction and archived using an automated DNA extraction technique at the Singapore Eye Research Institute (SERI). Extracted DNA samples are aliquoted and stored at −80◦C.<br><br>Furthermore 2 ml of plasma samples are aliquoted and stored at −80◦C for adiponectin testing.<br><br>A total of 50 ml midstream urine is collected to determine levels of micro	<u>Diabetes</u>: HbA1c >6.5%, random blood glucose ≥11.1 mmol/L, use of diabetic medication [79], or self-reported<br><u>Hyperlipidaemia</u>: High levels of total cholesterol (≥6.2 mmol/L) and/or use of anti-lipid medication<br><u>Dyslipidaemia:</u> High levels of total cholesterol (≥5.2 mmol/L) and/or low levels of HDL cholesterol (<1 mmol/L in men and <1.3 mmol/L in women), and/or self-report<br><u>Chronic kidney disease</u>*:* Estimated glomerular filtration rate (eGFR) <60mL/min/1.73m2, based on the US National Kidney Foundation Kidney Disease Outcome Quality Initiative (KDOQI) Working Group definition [80]. eGFR is estimated from the serum creatinine concentration

© 2020. Gupta P et al. Published online at http://www.aginganddisease.org/EN/10.14336/AD.2020.0206

		albuminuria and creatinine for calculation of the albumin-to-creatinine ratio.	(eGFR) [81] using the CKD Epidemiology Collaboration (CKD-EPI) equation.
**Ocular Tests**			
Auto-refraction and ocular biometry	Auto-refractor machine (Canon RK-5 Auto Ref-Keratometer, Canon Inc. Ltd., Japan).<br><br>A non-contact partial coherence laser interferometry (IOLMaster V3.01, Carl Zeiss; Meditec AG Jena, Germany)	A minimum of three readings are averaged to give a single estimate of refractive error.<br><br>We assess the participant's axial length (AL), anterior chamber depth (ACD), and corneal curvature (CC) in the horizontal and vertical meridian. Five consecutive readings, within 0.02 mm of each other and with a signal-to-noise ratio of ≥ 2.0, are taken to ensure consistency in AL measurement. Three measurements each are obtained for ACD and CC, respectively.	
Spectacle prescription	An automatic lensmeter (Nidek Auto- Lensmeter, model LM990; Nidek Co., Ltd., Japan)	Trained optometrists assess participants' habitual prescription (spherical, cylinder and axis).	
Distance Visual Acuity (presenting & best corrected) and subjective refraction	Early Treatment for Diabetic Retinopathy Study (ETDRS) LogMAR chart (Lighthouse Distance VA Number Chart CAT No. C102)	Monocular and binocular presenting distance visual acuities (PDVA) are assessed with the participant wearing his/her habitual prescription under photopic conditions (85 cd/m^2) at 4 m. If the participants are unable to read the largest line of letters on the VA chart, the chart is moved to 2 m. However, if he/she is still unable to make out any lines at 2 m, finger counting, hand movement and the ability of the eye to perceive light with a pen torch are assessed. Binocular measurements are made with both eyes viewing the target. Monocular and binocular best corrected distance VA (BCDVA) is then assessed using a trial frame and lenses under the same photopic conditions as that of PDVA measurements.<br><br>Using the autorefraction results as a starting point, the refinement of the sphere, cylinder and axis is performed until the BCDVA is obtained. Both PDVA and BCDVA are scored on a letter by letter basis with each letter worth 0.02log units.	Refractive error is determined by subjective refraction.<br>Myopia: spherical equivalent (SE) refraction <-0.5 diopters (D)<br>Hyperopia: SE >0.5 D<br>Astigmatism: cylinder <-0.5 D<br>Anisometropia: difference in SE >1.0 D<br>Under corrected refractive error: difference of ≥0.2 logMAR between PDVA & BCDVA<br><br>Visual impairment (VI) is defined according to the U.S definition as VA<20/40 to >20/200 (logMAR >0.3 to <1.00)<br><br>Blindness: defined as VA ≤20/200 (logMAR ≥1.00)
Near Visual Acuity	Near logMAR chart (Lighthouse International, New York, New York, USA).	Binocular presenting near VA (PNVA) is performed under photopic conditions at 40 cm with the participant's habitual correction. The same methodology for PDVA and BCDVA is used to assess PNVA, namely the last line attempted, where ≥ 3 mistakes are made, combined with the number of mistakes made on previous lines, are used to calculate a letter-by-letter logMAR PNVA score. Binocular best corrected near VA (BCNVA) measurement is conducted after BCDVA assessment, with participants' best corrected prescription for the testing distance. Plus sphere trial lenses are utilized	Presbyopia is defined as presenting near VA worse than N8 print (near logMAR<0.2).

© 2020. Gupta P et al. Published online at http://www.aginganddisease.org/EN/10.14336/AD.2020.0206

		to obtain BCNVA measurements binocularly. The spherical dioptric correction is recorded along with the corresponding BCNVA.	
Contrast Sensitivity	Pelli-Robson Contrast Sensitivity Chart	Contrast sensitivity (CS; ability to recognize targets of different levels of contrast (faintness)) is measured monocularly and binocularly with the participant's best distance correction, corrected for the testing distance of 1 m with a +0.75D working distance lens, under photopic conditions. Scores range from 0.00 to 2.25 logCS with higher values indicating better CS.	Contrast sensitivity impairment is defined as CS<1.55 logCS
Stereo-Acuity	The Frisby Stereo test [35]	For binocular stereo-acuity (ability to see in 3-D), participants are asked to identify the circle in one of four squares for each of the three plates at a distance of 60 cm while wearing best corrected near correction. Scores range from 40-150 arc sec with lower values indicating better SA. If they are unable to perform the test at 60 cm then the test distance is reduced to 30 cm. Participants who cannot identify the target at 30 cm are classed as stereo-negative.	Stereo-acuity impairment is defined as SA≥150 arc sec
Colour Vision	Farnsworth D-15 test	Participants are asked to arrange 15 different colour discs in a sequential colour series, monocularly, while wearing best corrected near correction.	Abnormal colour vision is defined as one or more major crossings, where the difference between two adjacent caps was more than three steps in the better-eye.
Binocular Central Motion Sensitivity	Computer-based motion sensitivity program	Motion sensitivity is assessed binocularly with habitual correction at a 3.2 m testing distance. Participants report the perceived direction of movement of a group of small targets generated on the computer screen (which move in one of four directions) and the minimum amount of stimulus movement that is correctly perceived is recorded as a displacement threshold, in logDegArc.	
Attentional Field of View (AFoV)	Computer-based field of view program	AFoV is a functional test of binocular visual processing speed for rapid detection and localization of central and peripheral targets under conditions of divided and selective visual attention (absence and presence of visual distractors respectively). The test assesses higher order cognitive abilities, but performance also relies on visual sensory function since targets must be visible in order to be detected. The test has demonstrated high levels of reliability and validity [36, 37]. The test was conducted on an iPad, where participants identify a central task (direction of a large high-contrast tumbling E) and simultaneously identify the location of a peripheral target (a low-contrast Gabor patch). Processing speeds are calculated as the minimum presentation time (in ms) at which participants correctly identify both the central and peripheral targets. Faster	

© 2020. Gupta P et al. Published online at http://www.aginganddisease.org/EN/10.14336/AD.2020.0206

		speeds indicate better visual processing speeds.	
Neuro-psychological test of Visual Attention Trail Making Test (TMT)	Trail-making A and B tests	Trail-making A and B tests are pen and paper tests which participants complete binocularly with their habitual near correction. In Part A, participants have to draw lines to connect in ascending order a series of randomly positioned circles containing numbers (1-2-3…25), as quickly as possible. In Part B, the circles include both numbers and letters, and participants have to draw lines to connect the circles in an alternating ascending order between the numbers and letters (1-A-2-B-..13-L), which requires additional executive function processing during the visual search task. The time to complete both tests is recorded in seconds.	The primary variables of interest are the total time to completion for parts A and B. A cut-off time of 300 sec is used to discontinue test administration and is therefore the typical maximum score [82].
Pre-dilation slit-lamp examination and intraocular pressure (IOP)	(Haag-Streit model BQ-900; Haag-Streit, Switzerland) and Goldmann applanation tonometer (Haag-Streit, Switzerland)	A trained ophthalmologist performs a structured slit-lamp biomicroscopy examination before pupil dilation to determine abnormalities in the anterior segment of the eye and assesses the intraocular pressure (IOP). Assessment of IOP follows a standardized protocol as described elsewhere [83]. One reading is taken from each eye and recorded. If the reading is >21 mmHg in any of the eye, then a repeat reading is taken, and average of the two readings is recored as the final IOP.	Glaucoma suspect: eyes are defined as 1) IOP greater than 21mm Hg; 2) vertical cup to disc ratio (VCDR) of >0.6 or VCDR asymmetry of >0.2; 3) abnormal anterior segment deposit consistent with pseudoexfoliation or pigment dispersion syndrome; 4) narrow anterior chamber angle; 5) peripheral anterior synechiae; 6) other findings consistent with secondary glaucoma; 7) a known history of glaucoma [84].<br><br>Glaucoma is defined according to the International Society for Geographical and Epidemiologic Ophthalmology (ISGEO) criteria [85].
Gonioscopy	Goldmann four-mirror lens	Gonioscopic classification of the angle width, iris angle insertion, iris profile, and presence or absence of peripheral anterior synechiae is done according to the classification system proposed by Spaeth [86] and Scheie [87].	
Visual fields test	Humphrey Visual Field Analyzer II (model 750, Carl Zeiss, Switzerland)	A 24-2 Swedish Interactive Threshold Algorithm-Fast (SITA-FAST) static, threshold-related visual field (VF) examination is performed with near refractive correction prior to dilation. Test reliability is determined by the instrument's algorithm (fixation losses <20%, false positives <33%, or false negatives <33%). The visual fields test is repeated once if deemed unreliable or abnormal. One in five non-glaucomatous participants are also randomly selected to undergo VF testing.	
Pupil dilation	Tropicamide 1% and phenylephrine hydrochloride 2.5% (1 drop each)	All participants have their pupils dilated, except for participants with a known allergy to mydriatic eye drops, closed anterior chamber angles, and/or with IOP greater than 21 mmHg. Participants with narrow anterior chamber angles are cautioned about acute angle closure symptoms prior to dilation, and have their IOP measured again prior to discharge.	
Fundus photography	A digital nonmydriatic retinal camera (Canon CR-DGi with a 10D	7 field colour photographs of Early Treatment for Diabetic Retinopathy Study (ETDRS) of each eye.	Diabetic Retinopathy (DR) is graded from fundus photographs using the modified Airlie House classification

© 2020. Gupta P et al. Published online at http://www.aginganddisease.org/EN/10.14336/AD.2020.0206

	SLR backing, Canon, Japan)		system and a modification of the Early Treatment Diabetic Retinopathy severity system for diabetic retinopathy [88]. Grading includes the presence/severity of diabetic macular edema and presence of laser treatment.<br><br>Age-Related Macular Degeneration (AMD): AMD lesions are graded from fundus photographs using the modification of the Wisconsin AMD classification [89].
Post-dilated slit lamp examination	(Haag-Streit model BQ-900; Haag-Streit, Switzerland)	The post-dilation examination includes an assessment of intraocular lens status (phakic/pseudophakic/aphakic), clinical grading of cataract (if any) and an optic disc and fundus examination using a slit lamp. All examinations are done using a 78 Diopter lens at X10 magnification and follows standardized clinical protocols [83, 85]. The vertical dimensions of the optic disc and cup are estimated using an eyepiece graticule etched in 0.1 mm units. Measurements of vertical disc diameter exclude areas of peripapillary atropy and Elschnig's ring. For small discs with no visible cup, the measurements are taken as the diameter of the emerging retinal vessels [83, 85].	The presence and severity of Cataracts or pseudophakic/aphakic status of the lens are graded using the Lens Opacities Classification System III (LOCS-III) [90].
Retinal Optical Coherence Tomography	The Cirrus high-definition optical coherence tomography (HD-OCT) (software version 6.0; Carl Zeiss Meditec, Inc, Dublin, California, USA)	Scan for macula, retinal nerve fiber layer (RNFL), and optic disc health are obtained.	
Optical Coherence Tomography Angiography	The Optovue Angiovue Essential™	Scans of macula and optic disc are captured to obtain retinal microvasculature and blood flow information.	
**Non-Ocular Tests**			
Gait Speed<br><br>Gait Signature		Walking speed is assessed with participants walking a distance of 4 m (15 feet) at their usual speed. Scoring of the gait speed test is in seconds.<br><br>Comprehensive and objective measurements of movement features, such as balance control, rhythmicity, coordination, and obstacle avoidance, are obtained using a ZurichMOVE Sensor. Participants are asked to walk for a minimum of 2.5 minutes to a maximum of 6 minutes, and their gait signatures/features are captured and analyzed by the Sensor.	Slowness is defined as a gait speed score of less than 0.8m/s [38].<br><br>Low grip strength is defined based on the Asian working group for Sarcopenia (AWGS) [38] as a grip strength <26 kg and <18 kg, in men and women, respectively.<br><br>Sarcopenia is defined based on the AWGS [38]- low muscle mass (Men <7 kg/m^2, Women <5.4 kg/m^2) + low grip strength (Men <26 kg, Women <18 kg)
Hand grip strength	Digital hand dynamometer (Jamar Plus+).	Participants' grip strength (in kg) in the dominant hand is measured three times, with the participant seated and elbow flexed	

© 2020. Gupta P et al. Published online at http://www.aginganddisease.org/EN/10.14336/AD.2020.0206

		at 90 degrees, with a rest period of 30 seconds between each measurement. The average of the three readings is utilized in analyses.	or low muscle mass + slow gait speed (≤0.8 m/s)<br><br>Osteoporosis is defined as a low BMD (T-score, ≤ −2.5) in any of the following sites: hip, femoral, neck, and lumbar spine [91].<br><br>Sarcopenic obesity is considered present when sarcopenia and obesity are both evident.<br><br>Osteosarcopenia is considered present when osteoporosis and sarcopenia is encountered in the same individual.<br><br>Osteosarcopenic obesity is considered present when osteoporosis and sarcopenia is encountered in the same individual.
Body composition & bone mineral density assessment	Dual energy X-ray absorptiometry (DXA; Hologic Discovery-W; Hologic Inc, Bedford-MA).	DXA, a non-invasive imaging modality utilising very low dose X-rays (~0.005 to 0.01 millisievert) is used to quantify participants' body composition (fat mass, and lean mass) and bone mineral density (BMD for entire single hip, and lumbar spine) measures. A trained radiographer goes through each scan to ensure accuracy and validity of the images.	
Physical activity and sleep monitor	ActiGraph GT9X	The participant is instructed to wear the ActiGraph watch on their non-dominant hand over a period of 7 consecutive days (24 hours a day) and maintain a sleep log. Raw acceleration data, number of steps taken, physical activity intensity, sleep latency, total sleep time and sleeping efficiency data are retrieved from the watch.	
Frailty	Fried or Hopkins frailty[53] phenotype.	*Unintentional shrinking* is defined as BMI <18.5 kg/m^2 and/or self-reported unintentional weight loss of 4.5 kg or more in the past 6-12 months.<br>*Slowness* is assessed with a habitual gait speed test (described above).<br>*Weakness* is determined using the hand grip strength test (described above). We will use Singapore specific cut-offs for hand grip strength. A grip strength of <31.1 kg (men) and <18.2 kg (women) in the age group 60-64 years, and <18.5 kg for men and <12.4 kg for women in the oldest age group (85+ years) is considered as muscle weakness [92].<br>*Exhaustion* is measured with 3 questions related to the vitality domain from the 12-item Short form survey (SF-12) used in the Medical Outcomes Study: "Did you feel worn out?" "Did you feel tired?" "Did you have a lot of energy?" The questions are rated on a five-point Likert scale ranging from 'All of the time' to 'None of the time'. The scores are summed, a higher score indicating more energy, and a score of < 10 denoting exhaustion.<br>*Low physical activity* is assessed based on self-reported time (in hours) spent doing light (e.g., office work, driving a car,	Physical Frailty: The Fried or Hopkins frailty [53] phenotype is used to define physical frailty. The tool defines the frailty phenotype as meeting 3 or more of the 5 criteria, pre-frailty as having 1 or 2 of these characteristics, and not frail as having none.

© 2020. Gupta P et al. Published online at http://www.aginganddisease.org/EN/10.14336/AD.2020.0206

		strolling, standing with little motion, personal care), moderate-vigorous activities (e.g., gardening, brisk walking, dancing, jogging, swimming, strenuous sports). The total amount of time spent performing moderate and vigorous activities per week and activity time below the gender-specific lowest quintile is used to denote frailty.	
**Sensory Assessment**			
Functional hearing	Portable audiometer (SHOEBOX Audiometry, Ontario-Canada)	Functional hearing is quantified in a quiet room. Four frequencies are assessed (500, 1000, 2000, 4000 hertz) and the average of the hearing thresholds is taken.	Hearing impairment is defined as an average threshold of ≥25 dB in the better-hearing ear [93].
Smell identification	Sniffin' Sticks test [94]	Participants are asked to identify 16 different odours (e.g. coffee, shoe leather, orange) from felt pen tips impregnated with 4 ml of the odourant at intervals of 30 seconds between each odour presentation, and tasked to choose the correct answer from four different options presented to them in the form of multiple choice questions. The number of correct answers was then summed to give an overall score.	Smell disorder is defined based on normative value for the identification test as per the manufacturer guidelines. The score range from 0 to 16. Score of 0 to <8 represent anosmia (inability to perceive smell); 8-11 demonstrate hyposmia (reduced ability to smell), and ≥12 indicate normosmia (normal olfactory function). Based on above, a score of <12 will be considered some form of smell disorder.
Oral health		A thorough oral health examination is conducted to assess oral health conditions (i.e. dental caries, periodontal disease, and denture status).<br><br>Chewing ability and dry mouth symptoms are also assessed. Chewing efficiency is evaluated using a colour-mixing ability test [95]. Participants are asked to chew strips of commercially available two-coloured chewing gums for 20 chewing cycles and the bolus is subsequently retrieved, scanned and analysed opto-electronically.<br><br>Stimulated and unstimulated whole saliva is collected for dry mouth (xerostomia) assessment.	DMFT index will be used to numerically express the dental caries prevalence and will be obtained by calculating the number of Decayed (D), Missing (M), Filled (F) teeth (T) in an individual. Patients with higher DMFT have higher dental caries rate and tend to have poorer oral health status.<br><br>Full mouth bleeding score (FMBS) will be used for the evaluation of periodontal conditions. This score records the extent of gingival bleeding as a result of inflammation. Each tooth present is gently probed with a periodontal probe at six sites. The number of sites where bleeding is recorded is divided by the total number of available sites in the mouth and multiplied by 100. Individuals with low mean FMBS (< 10% of the surfaces) will be regarded as patients with a low risk for recurrent disease, while those with FMBS > 25% will be considered to be at high risk for periodontal breakdown [96].

© 2020. Gupta P et al. Published online at http://www.aginganddisease.org/EN/10.14336/AD.2020.0206

			Digital scanning of the two-colour chewing gum test specimen provides reliable quantitative data for chewing efficiency. If the colours of the specimen are not mixed, two well-separated peaks on the hue axis are present. With increasing degree of colour mixing, the two hue peaks of each colour group converge and will eventually fuse at an intermediate position into one peak when the colours are perfectly mixed.The variance of the hue (VOH) is considered as the measure of mixing. Hence, inadequate mixing presents with larger variance on the hue axis than complete mixing.<br><br>Reduced salivary secretion is the most important cause of xerostomia and this can adversely affect the oral health condition, resulting in higher caries rate. The normal flow rate for unstimulated, “resting” whole saliva is 0.3 to 0.5 mL/min; for stimulated saliva, 1 to 2 mL/min. Values less than 0.1 mL/min are typically considered xerostomic.

© 2020. Gupta P et al. Published online at http://www.aginganddisease.org/EN/10.14336/AD.2020.0206
